# Conservative Treatment of Carpometacarpal Dislocation of the Three Last Fingers

**DOI:** 10.1155/2016/4962021

**Published:** 2016-09-14

**Authors:** Hélène Jumeau, Philippe Lechien, Florence Dupriez

**Affiliations:** Emergency Department, Jolimont Hospital, 159 Ferrer Road, 7100 Haine Saint Paul, Belgium

## Abstract

Posterior carpometacarpal (CMC) dislocation is a rare condition. Treatment is usually surgical though no strict consensus can be found upon literature review. If diagnosed early and no associated fractures are found, CMC dislocation could benefit from conservative treatment comprising closed reduction and splint immobilisation. We report the case of a 26-year-old man diagnosed with a posterior dislocation of the third, fourth, and fifth CMC joints after a fall of 1.5 meters, treated by external reduction under procedural sedation and immobilisation with a cast for 6 weeks. Evolution was excellent with no relapse observed during follow-up. Our aim is to increase physician awareness of CMC dislocation so that they seek this injury in the emergency department. Unrecognised CMC dislocation can lead to neurovascular injuries as well as chronic instability and early articular degeneration.

## 1. Introduction

Carpometacarpal (CMC) dislocation is a rare condition usually treated surgically [1, 2]. Posterior dislocation is more common (85%) than palmar dislocation [3]. Most posterior dislocations are due to high velocity trauma. Delayed treatment can result in neurovascular injuries due to oedema [3] and prolonged compression. Untreated, these lesions can result in chronic instability of the CMC joints and early articular degeneration [4]. We report the case of a 26-year-old man suffering from a posterior dislocation of the third, fourth, and fifth CMC joints after a fall of less than 1.5 meters, treated conservatively.

## 2. Case Presentation

**Figure 1:**
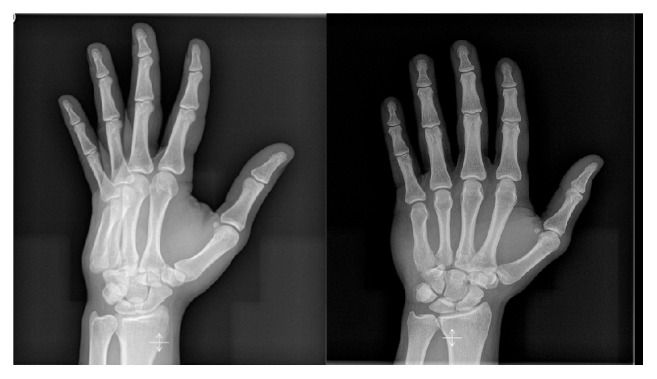
X-Ray: oblique and anteroposterior view of the hand. Oblique view shows a complete dislocation of the fourth and fifth CMC joints and a partial dislocation of the third CMC joint.

**Figure 2:**
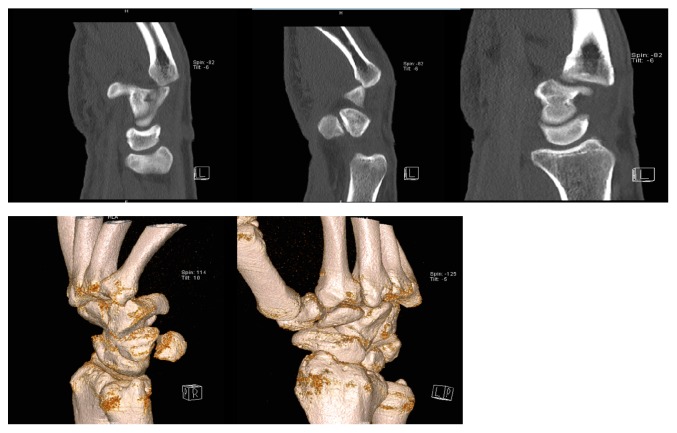
Complementary CT scan showing no other lesions than complete dislocation of the fourth and fifth CMC joints and a partial dislocation of the third CMC joint.

**Figure 3:**
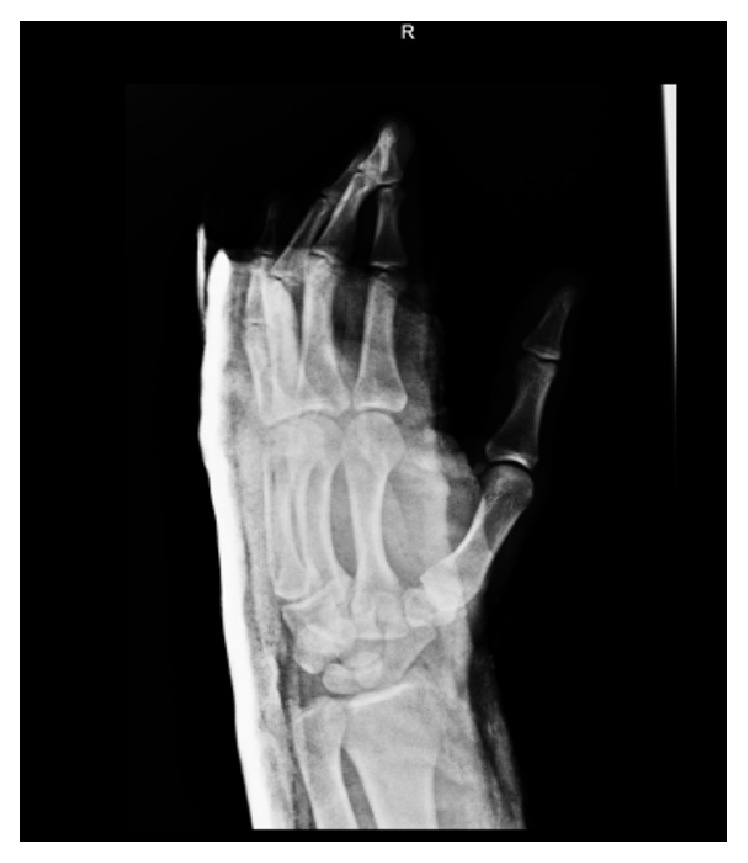
After reduction X-ray: optimal reduction, no more signs of dislocation.

A 26-year-old man, with no significant medical history, suffered from a posterior dislocation of the third, fourth, and fifth CMC joints after the patient stumbled and fell on his outstretched right hand. The patient presented rapidly to our emergency department with a swollen hand and complaining of acute pain. He was unable to move his wrist and kept the hand in a neutral position. Clinical examination showed posterior tumefaction of the right hand with no wound. No distal neurological nor vascular impairment was observed. Motor integrity of the fingers was preserved but revealed slight malrotation. Systemic complete examination showed no additional lesions. Despite a normal anteroposterior X-ray of the hand, an oblique view (Figure 1) showed a complete dislocation of the fourth and fifth CMC joints and a partial dislocation of the third CMC joint with no associated fractures. CT scan was performed showing no additional lesions (Figure 2). After discussion with the hand surgeon, the decision was made to reduce the dislocation in the emergency room under procedural sedation (midazolam 0.03 mg/kg associated with ketamine 1 mg/kg). Applying a longitudinal traction to the involved fingers with an associated pressure over the base of the dislocated metacarpals accomplished reduction. Examination after reduction showed correction of the malrotation. The wrist was then immobilised in a palmar splint from midforearm to the third phalanx of all fingers except the thumb, with slight dorsiflexion of the wrist. Control oblique X-ray of the hand showed adequate alignment of the CMC joints (Figure 3). Radiographs of the hand were performed during follow-up to ensure the absence of relapse. After six weeks of conservative treatment, clinical control showed no recurrence of CMC instability nor reduced strength of the hand. A 6-month follow-up did not show chronic pain of the hand.

## 3. Discussion

CMC joints are usually stable thanks to strong transverse dorsal CMC ligaments and longitudinal volar CMC ligaments [5]. Most dislocations occur after high-energy traumatisms [6]. These lesions are often underdiagnosed in the emergency department due to the fact that patients suffering from such lesions usually present to the emergency department with other more obvious traumatisms [7]. In our case, the single traumatism of the hand made the diagnosis easier.

The frequency of posterior CMC dislocation is higher than that of palmar dislocation [7]. CMC joint dislocation represents less than one percent of all hand trauma, the first CMC joint excluded [1, 7]. The dislocation of the second and the third CMC joints is even less frequent [8].

When CMC dislocation is suspected based on clinical findings (pain, swelling, and lump of the joints), anteroposterior, profile, and oblique X-rays of the hand should be performed [9]. Usually diagnosed with a true lateral view X-ray of the hand, CMC dislocation can be concealed due to overlapping of the joints [9]. On posteroanterior radiographs, such dislocations can be suspected when loss of parallelism between CMC joints is found or when an apparent shortening of metacarpals is noticed [9]. Additionally, oblique radiographs of the hand can be useful to demonstrate CMC dislocation [2]. In our case report, oblique X-rays led to the diagnosis. Associated fractures of the hand and the wrist have to be excluded with certainty in order to propose adequate treatment. To do so, a CT scan should be performed [10]. Regularly, carpal fractures are occult on conventional X-rays. Surgical treatment is strictly recommended if an associated fracture is found [10].

Mostly, CMC dislocations are treated surgically [1] either by open reduction and internal fixation or by closed reduction and percutaneous pinning. Few cases of closed reduction and conservative treatment with splint immobilisation are reported in literature [2]. In our case, the patient had no other lesion than CMC dislocation on the CT scan and showed an excellent outcome after six weeks of splint immobilisation. A failed treatment would be assessed by relapse of dislocation, residual pain, or limitation in finger movements and diminished strength of the hand [10]. In the case of imprecise alignment or chronic dislocation, frequent complications include posttraumatic arthrosis, median nerve dysfunction, carpal instability, complex regional pain syndrome, and tendon problems. In our patient, none of these were found after follow-up.

Some secondary dislocations after treatment by closed reduction and splint immobilisation have been described, occurring within two weeks of the reduction [2]. Therefore, X-rays of the hand are recommended during follow-up. Surgical closed reduction treatment shows good results if undertaken within 10 days of the dislocation [6]. A CMC dislocation diagnosed early could therefore benefit from a conservative closed reduction under procedural sedation with splint immobilisation. Percutaneous reduction could be considered if a recurrence was found at follow-up within the first ten days.

After three weeks of evolution without treatment, a surgical reduction is strongly recommended [6].

CMC can easily be underdiagnosed if clinical signs are overlooked. We hope that CMC dislocation will no longer be an underestimated lesion in daily practice and that more closed reduction followed by conservative treatment will be practiced successfully. Physicians should consider CMC dislocation in every patient presenting with hand trauma.
